# Administrative health data in Canada: lessons from history

**DOI:** 10.1186/s12911-015-0196-9

**Published:** 2015-08-19

**Authors:** Kelsey Lucyk, Mingshan Lu, Tolulope Sajobi, Hude Quan

**Affiliations:** Department of Community Health Sciences, Cumming School of Medicine, University of Calgary, 3280 Hospital Dr. NW, Calgary, Alberta T2N 4Z6 Canada; Department of Economics, University of Calgary, 2500 University Dr. NW, Calgary, Alberta T2N 1 N4 Canada; O’Brien Institute for Public Health, Cumming School of Medicine, University of Calgary, 3280 Hospital Dr. NW, Calgary, Alberta T2N 4Z6 Canada

**Keywords:** Administrative data, Health services research, Health informatics, History

## Abstract

**Background:**

Health decision-making requires evidence from high-quality data. As one example, the Discharge Abstract Database (DAD) compiles data from the majority of Canadian hospitals to form one of the most comprehensive and highly regarded administrative health databases available for health research, internationally. However, despite the success of this and other administrative health data resources, little is known about their history or the factors that have led to their success. The purpose of this paper is to provide an historical overview of Canadian administrative health data for health research to contribute to the institutional memory of this field.

**Methods:**

We conducted a qualitative content analysis of approximately 20 key sources to construct an historical narrative of administrative health data in Canada. Specifically, we searched for content related to key events, individuals, challenges, and successes in this field over time.

**Results:**

In Canada, administrative health data for health research has developed in tangent with provincial research centres. Interestingly, the lessons learned from this history align with the original recommendations of the 1964 Royal Commission on Health Services: (1) standardization, and (2) centralization of data resources, that is (3) facilitated through governmental financial support.

**Conclusions:**

The overview history provided here illustrates the need for longstanding partnerships between government and academia, for classification, terminology and standardization are time-consuming and ever-evolving processes. This paper will be of interest to those who work with administrative health data, and also for countries that are looking to build or improve upon their use of administrative health data for decision-making.

## Background

Many health researchers use administrative data (e.g., physician billing claims) to inform and enhance decision-making and patient outcomes [1]. In the health care system, for example, administrative health data may be used to regulate patient flow, determine resource-use, or distribute funds to hospitals.

Canada has some of the most comprehensive and high-quality administrative health data in the world, in part due to its universal health insurance registries, comprehensive coverage of inpatient and outpatient services, and linkage of databases via unique personal identifiers within provinces and territories [2]. The largest administrative health database in Canada is the Discharge Abstract Database (DAD), a national database that contains administrative, clinical, and demographic information relating to all separations from acute care institutions in Canada [3]. As of 2002, the DAD represented approximately 75 % of all inpatient discharges in Canada [4]. The DAD has constituted a founding databases of the Canadian Institute for Health Information (CIHI) since 1994, and has contributed to the high quality state of administrative health data in Canada that exists today.

Underlying this current state of administrative health data lays the rich and relatively unknown history of this field and the factors that have led to some of Canada’s successes. A summary of key events is provided in Table 1. The purpose of this paper is to provide an historical overview of Canadian administrative health data for health research to contribute to the institutional memory of this field and inform present-day initiatives that aim to improve health information and enhance decision-making. We conclude our paper with recommendations for present-day research in administrative health data, which are drawn from the historical lessons that emerged in our analysis. This paper will be of interest to those who work with administrative health data and also those in other countries that are looking to build or improve upon their administrative health data.

**Table 1:** Recommendations for Health Statistics, as proposed in the *Hall Report*, 1964

Recommendations:	
1) federal financial support in consolidating existing registers;	
2) incorporating of modern data processing methods to analyze statistics;	
3) standardize classification of diseases, and social and demographic characteristics to facilitate comparability between regions; and,	
4) encourage timely publication of statistics and reports; and finally,	
5) establish a national clearing house and coordinating agency for health statistics [12].	

From: *Hall E. Royal Commission on Health Services. Ottawa: Queen's Printer,1964*

## Methods

We searched the academic and grey literature (e.g., PubMed, Google) for histories related to administrative health data, by using key words such as “health information,” “health statistics,” “administrative data,” and “history.” As we gained familiarity with our sources, we included more specific terms to identify additional sources (e.g., “Manitoba Centre,” “hospital morbidity database”). We used snowball sampling to further identify relevant research centre histories, individual biographies, and websites from provincial and national data centres from documents referenced by our sources. For brevity, we identified approximately 20 publicly available key sources (from ca. 80) that encapsulated historical trends in Canadian administrative health data. We conducted a qualitative content analysis of these sources, informed by historical methodology [5], following the methods put forth by Krippendorf. [6] According to Krippendorf, texts are considered artifacts of a given social and cultural context that affects how they are written, read, and interpreted. After a close reading of each source, we extracted content related to the events, individuals, challenges, and successes that emerged as pertinent to the history of administrative health data in Canada. We then compiled our findings to construct an historical narrative, spanning from 1847 to the present day (2015).

## Results and discussion

### A brief history of health statistics in Canada (1847–1964)

The collection of health-related statistics in Canada formally began in 1847 when the *Census and Statistics Act of 1847* mandated the collection of vital statistics and information regarding land holdings, dwellings, and demographics [7, 8]. Initially, the census was a responsibility of the provinces, but became a federal responsibility when the country unified in 1867 [9]. This initiated the long-standing trend in Canadian health statistics of provinces reporting data to federal sources for compilation, analysis, and publication [7].

Two individuals are particularly important during this early history of health statistics. First, former Deputy Minister of Agriculture, Joseph Charles Taché (1820–1894), standardized the collection of information by census-takers, which allowed for comparable analysis across provinces for the first time [9]. Second, former Minister of Finance, Arthur Harvey (1834–1905), voluntarily collected and compiled various types of administrative statistics from government departments (e.g., hospitals receiving government grants, coroners’ inquests), which he published and sold to Canadians as the *Year Book and Almanac of Canada* [10]. Harvey’s *Almanac* was formally legislated and transferred to Taché’s Department in 1879 and published as the *Statistical Abstract and Record*. [10]

Throughout the 20th Century, statistical operations in Canada became centralized. With cooperation from 8 provinces, a national system of vital statistics was established,^1^[8] with all provinces and territories involved by 1949 [8]. In 1948, former Dominion Statistician, Herbert Marshall (1888–1977), amended the *Statistics Act* to give the federal government authority to “collect, compile, analy[z]e, abstract and publish statistics” to include “hospitals, mental institutions, tuberculosis institutions, and charitable and benevolent institutions.” [11] However, while this was meant to provide timely and informative statistics, the implementation of Marshall’s *Act* was far from efficient.

As would later be identified in the 1961–1964 Royal Commission on Health Services (RCHS), the lack of communication between the Dominion Bureau of Statistics and other departments throughout the first half of the 20th Century resulted in the timely and often duplicated collection of statistics [11]. Justice Emmett Hall (1898–1995), Chair of the RCHS, identified in 1964 that Canada’s “main need was for national statistics and for data showing significant variations among provinces and regions,” and recommended a national, federally-financed clearinghouse be established to facilitate this [11]. The RCHS identified a number of solutions to improve the state of health statistics in Canada, which are listed in Table 2.

**Table 2:** Timeline of key events in the history of administrative health data in Canada

Date	Event
1847	*Census and Statistic Act of 1847*
1879	*Year Book and Almanac of Canada* statistics transferred to Department of Agriculture
1884	Bureau of Labour Statistics established
1885	Ontario and Québec hospitals begin collecting hospital data
1918	Dominion Bureau of Statistics established
1921	Bureau establishes system of vital statistics for 8 provinces
1926	Québec, the Yukon and Northwest Territories join the national system of vital statistics program
1948	*Statistics Act* amended to give Bureau authority to collect hospital data
1949	Newfoundland joins Canada, and its vital statistics program
1960	Bureau establishes Hospital Morbidity Database
1961	Royal Commission on Health Services commences
1963	Hospital Medical Records Institute (HMRI) established
1964	Royal Commission on Health Services ends
1970	HMRI expands to 5 provinces
1977	HMRI become not-for-profit, includes standardized classification
	Rooses publish the first paper in Manitoba using administrative health data
1983	HMRI develops Case Mix Groups
1988	British Columbia Linked Health Data Project (BCLHDP) begins
1990	Canadian Centre for Health Services and Policy Research (CHSPR) established
1991	HMRI collects information for 75 % of discharges from Canadian hospitals
	Institute for Clinical Evaluative Sciences established
	Manitoba Centre for Health Policy established
	National Task Force on Health Information recommends a coordinating council for health information
1994	Canadian Institute for Health Information established
	HMRI holdings transferred to CIHI
1996	CHSPR permitted access to BCLHDP data
2002	Discharge Abstract Database covers 75 % of all inpatient discharges, for all provinces and territories (except QUE)
2005	ICD-10-CA and Canadian Classification of Health Interventions implemented by CIHI
2008	Population Data BC established
2009	CHSPR transfers data holdings to Population Data BC
2012	20^th^ Anniversary of ICES and MCHP

Legend: HMRI is Hospital Medical Records Institute; BCLHDP is British Columbia Linked Health data Project; CHSPR is Canadian Centre for Health Services and Policy Research; MCHP is Manitoba Centre for Health Policy; CIHI is Canadian Institute for Health Information; DAD is Discharge Abstract Database; QUE is Québec; ICD-10-CA is International Classification of Diseases, 10th revision, Canadian modification; ICES is Institute for Clinical and Evaluative Sciences; BC is British Columbia

### Recent developments in the history of administrative health data: HMRI and CIHI

In 1963, the Hospital Medical Records Institute (HMRI) was established to assist with the administration of provincial and federal hospital insurance plans in 1963 [12]. The Institute evaluated and compared the quality of medical care of hospitals and assisted provincial/federal insurance plans by compiling statistics from patient charts [12]. While initially intended only for Ontario, by 1970 the HMRI included 77 general hospitals in 5 provinces [12]. By 1977, the HMRI received 1.5 million chart abstracts per year, which grew to 4.1 million by 1991 [13].

The HMRI is important to the history of administrative health data particularly due to the methodological innovations it brought to this field. It encouraged the standardized coding of diseases and operations in accordance with the International Classification of Diseases, *Adapted for Indexing Hospital Records* (ICDA) among its participating institutions [12]. In 1983, the HMRI further increased comparability of health information with their introduction of *Case Mix Groups* (CMGs) [13]. CMGs allowed for comparisons between patient groups with similar resource-use or diagnoses, and estimations of length of stay and expected cost of care [13]. Eventually, the HMRI was transferred to CIHI to form a founding database of the DAD, in 1994.

The Bureau of Statistics also implemented a collection program for health information beginning in 1960—the Hospital Morbidity Database, which obtained data from provincial ministries of health [14]. However, the provinces favoured the services of HMRI’s centralized data processing system (compared to the Bureau’s edits for comparability), which led to its success [14].

In 1991, the National Health Information Council (NHIC), Conference of Deputy Ministers of Health, and Chief Statistician held a national inquiry into the state of health information [15]. At the time, four independent groups were responsible for health information, nationally: (1) the Canadian Centre for Health Information—a division of Statistics Canada; (2) the HMRI; (3) the Management Information Standards Group; and, (4) Health and Welfare Canada [15]. Thus, the NHIC recommended the establishment of a national coordinating council that represented non-government, government, and private sector organizations [15]. Eventually, this resulted in the 1994 founding of CIHI, implemented under the direction of the Health Infrastructure Consortium of Canada. CIHI formed large national databases by combining health information from the organizations listed above. CIHI currently maintains 27 pan-Canadian databases, and provides education, reporting tools, and strategies for its users [4]. It exists through partnerships with the federal government, and partners with all provincial and territorial Ministries of Health [16].

#### Provincial centres for administrative health data research

Three existing provincial health research centres have been pivotal to the development and history of administrative health data research in Canada. This section will briefly review the histories of centres in Ontario, Manitoba, and British Columbia (BC).

### The Institute for Clinical Evaluative Sciences (ICES)

During the 1980s, the Ontario Medical Association (OMA) and Ministry of Health became interested in establishing a research centre that operated at arm’s length of the government to promote evidence-based health care [17]. David Naylor and Jack Williams of the Clinical Epidemiology Unit and Research program at Toronto responded in 1991 with a proposal for ICES, which would use administrative health data from the Ministry of Health for population-level research [18]. Their proposal was strengthened by the Ontario Health Insurance Plan’s recent issue of health cards to residents, which would allow for patients to be tracked by unique identifiers (e.g., age, medication) [18]. The proposal was quickly funded with a $20-million commitment from the Province [17].

As inaugural director, Naylor operated ICES as a scaled-up version of the multidisciplinary model (i.e., epidemiology, health services research, and policy formation). He implemented ICES at the Sunnybrook Hospital in Toronto, with a broadened scope to include academics, providers, administrators, and policy makers [17]. Initial goals of ICES were to investigate rates of medical procedures, length of stay, and drug administration [17]. The Province, OMA, and independent researchers and stakeholders each contributed one third to the research endeavors of ICES [17]. In its first year of operation, ICES published approximately 30 research papers, and developed a health atlas of system-related and disease-specific information [17].

Today, ICES holds de-identified, linkable data on 13 million of Ontario’s residents, under the Personal Health Information Protection Act, has 4 satellite sites in medical schools across Ontario and established remote access and analysis system for users [18–20]. ICES continues to operate within arm’s length of the Ontario Government and partners with numerous national/provincial programs to produce work that informs policy and expands the capacity for research in Canada [19].

### The Manitoba Centre for Health Policy (MCHP)

In Manitoba, the history of administrative health data research intertwines with the careers of Noralou and Leslie Roos. In 1973, the Rooses came to the University of Manitoba, where Noralou collaborated with David Fish of Community Health Sciences to project provincial healthcare needs using the administrative files of the Manitoba Health Services Commission (MHSC) [21]. The MHSC collected data on registration, hospital, Personal Care Home, and medical claims. [21] Initially, MHSC data was provided to Noralou on the basis of good faith and mutual trust, but eventually MHSC and Noralou devised a system where multiple data sources were linked to individuals, who could be followed through the services they sought. Linkage overcame limitations of MHSC data, such as not tracking births, deaths, or moves out of Manitoba [21]. With former Assistant Executive Director to the MHSC, Paul Henteleff, the Rooses published the first paper using linked administrative health data in Manitoba.^2^

In 1988, Fraser Mustard (former President of the Canadian Institute for Advanced Research) recruited the Rooses for his Population Health Program, and brought them together with the Provincial Government and Department of Medicine to form the MCHP. [21, 22] Together an agreement was reached where the MCHP would answer 6 research questions for the Province each year in exchange for funding, and otherwise be granted academic freedoms. [21] In 1990, the MCHP was given a 3-year contract for $3.2 million to encourage its operation within arm’s reach of the Province [21].

Today, the Centre employs over 60 persons in the fields of administration, communications, finance, data analysis, information technology, repository access, data acquisition, and research [23]. The Centre operated under the direction of Noralou until 2006, whose contribution to healthcare has recognized nationally with an Order of Canada [21, 22]. Today, the MCHP collaborates with researchers and institutions across Canada, and facilitates access to over 20 health databases, 5 social databases, 5 education databases, 4 registries, and 9 special data files in Manitoba [23].

### Population Data British Columbia (PopDataBC)

In BC, administrative health data research began with the establishment of the University of British Columbia’s (UBC) Health Sciences Centre. When the Centre first opened in the 1960s, the Health Services Research and Development unit was established and made partnerships with the BC Ministry of Health Services to maintain and provide access to health data throughout the 1970s [24].

Morris Barer and Robert Evans were two individuals pivotal to BC’s development of administrative health data research. In 1988, Barer, with colleagues from UBC, BC Cancer Agency, and BC Ministry of Health—began the BC Linked Health Data Project (BCLHDP), which “was designed to capture the power of linkable and longitudinal data, and avoid the inefficiencies associated with project-by-project data linkage.” [24] BCLHD included data from vital events and health care services utilized by nearly all BC residents from 1985, and today includes data from other agencies such as WorkSafeBC [24]. With Evans, who in 1990 was a member of the Royal Commission on Health Care and Costs in BC, Barer founded the UBC Centre for Health Services and Policy Research (CHSPR) within the UBC College of Health Disciplines [25]. CHSPR developed from the idea that UBC should influence health care policy and planning for BC [25]. Following an agreement with the BC Minister of Health and Privacy Commissioner in 1996, CHSPR was permitted to use BCLHD for applied health services and policy research [24, 25].

CHSPR obtained national funding to expand its breadth in data research in the 2000s, which allowed them to partner with UBC and other researchers from across the province to create PopDataBC: a “multi-university, data and education resource facilitating interdisciplinary research on the determinants of human health, well-being and development.” [24] Today, PopDataBC provides researchers with remote access to individual-level linked data on BC’s residents across the sectors of early childhood, workplace, environment, and more [24].

## Conclusions

The success of the increasingly expansive, standardized, and collaborative history of administrative health data in Canada reflects the recommendations made by the RCHS over 50 years ago. We echo these recommendations for the present field of administrative health data research, with lessons drawn from this history.

### Recommendation 1: Federal financial support in consolidating existing registers

At the federal level, financial support has been imperative to identifying national needs for health research. The founding of CIHI and NHIC was made possible through federal investments that improved the capacity for evidence-based decision-making. Likewise, support from provincial governments has also proven essential to the long-term sustainability of data centres. As shown for MCHP, for example, the support provided by the Province of Manitoba has led to a long-term collaborative working partnership.

### Recommendation 2: Standardize classification of diseases and social and demographic characteristics to facilitate comparability between regions

As shown throughout the history of administrative health data in Canada, the standardization of classification methods has been no easy task. As historical developments have shown (e.g., introduction of CMGs by the HMRI), standardization allows for comparisons, increases data quality, and reduces the need to adapt multiple versions of reported data. Standardization also facilitates the timely publication of reports, which are especially important when considering the increasing constraints to the resource capacity of data centres.

### Recommendation 3: Establish a national clearinghouse and coordinating agency for health statistics

CIHI and provincial data centres place Canada as a global leader in the field of administrative health data science. With government support, Canada’s administrative health databases have developed into innovative, high-quality, and informative resources for health research. As we have shown throughout this paper, the goals of establishing a national and standardized database and clearinghouse were realized long before its official founding in 1994. Provincial centres in Manitoba, Ontario, and BC have shown how smaller-scale developments can facilitate larger-scale project.

Evidence-based decision-making relies on high quality health information. This paper has provided an overview of the history of administrative health data in Canada, and has provided lessons learned from the past for present-day research. Specifically, we have highlighted the following recommendations from the 1964 RCHS: (1) standardization; (2) centralization of data resources; and, (3) government financial support. The short histories provided here illustrate the need for longstanding financial commitments, and collaboration between government and research centres in overcoming barriers to standardization and centralization (e.g., top-down decision-making).

## Abbreviations

BC: British Columbia
BCLHDP: British Columbia Linked Health Data Project
CHSPR: Centre for Health Services and Policy Research
CIHI: Canadian Institute for Health Information
CMGs: Case Mix Groups
DAD: Discharge Abstract Database
HMRI: Hospital Medical Records Institute
ICDA: International Classifications of Diseases, Adapted for Indexing Hospital Records
ICES: Institute for Clinical Evaluative Sciences
MCHP: Manitoba Centre for Health Policy
NHIC: National Health Information Council
OMA: Ontario Medical Association
PopDataBC: Population Data British Columbia
QUE: Québec
RCHS: Royal Commission on Health Services
UBC: University of British Columbia

## Footnotes

1 Prince Edward Island, Nova Scotia, New Brunswick, Ontario, Manitoba, Saskatchewan, Alberta, and British Columbia.

2 Roos, NP, Henteleff PD, and LL Roos. “A New Audit Procedure Applied to an Old Question: Is the Frequency of T&A Justified?” *Medical Care* 15 (1977):1–18.

## References

[1] Manitoba Centre for Health Policy. Administrative health data. http://umanitoba.ca/faculties/health_sciences/medicine/units/community_health_sciences/departmental_units/mchp/resources/repository/health_admin.html (2013). Accessed September 2014.

[2] Quan H, Smith M, Barlett-Esquilant G, Johansen H, Tu K, Lix L (2012). Mining administrative health databases to advance medical science: Geographical considerations and untapped potential in Canada. Can J Cardiol.

[3] Canadian Institute for Health Information (2012). Data quality documentation, Discharge Abstract Database—Multiyear information.

[4] Canadian Institute for Health Information. Discharge Abstract Database Metadata. http://www.cihi.ca/cihi-ext-portal/internet/en/document/types+of+care/hospital+care/acute+care/dad_metadata (2014). Accessed September 2014.

[5] Howell M, Prevenier W (2001). From reliable sources: An introduction to historical methods.

[6] Krippendorf K (2004). Content analysis: An introduction to its methodology.

[7] Statistics Canada. History of vital statistics. http://www.statcan.gc.ca/health-sante/vital/2012001/hvs-eng.htm (2012). Accessed September 2014.

[8] Statistics Canada. Vital statistics – Death database. http://www23.statcan.gc.ca/imdb/p2SV.pl?Function=getSurvey&SDDS=3233&Item_Id1635&lang=en. (2009). Accessed September 2014.

[9] Worton D (1998). The Dominion Bureau of Statistics: A history of Canada’s central statistical office and its antecedants, 1841–1972.

[10] Statistics Canada. The start of the *Canada Year Book*. http://www65.statcan.gc.ca/acyb07/acyb07_0001-eng.htm (2009). Accessed September 2014.

[11] Hall E (1964). Royal Commission on Health Services.

[12] Robertson CM (1970). The Hospital Medical Records Institute. Med Care.

[13] Yungblut RM (1991). Managing information and the Hospital Medical Records Institute.

[14] Statistics Canada. History of diagnosis and procedure classifications, Hospital Morbidity Database, Survey number 3203. http://www23.statcan.gc.ca/imdb-bmdi/document/3203_D6_T9_V1-eng.pdf. Accessed September 2014.

[15] National Task Force on Health Information. Health information for Caanda: Report of the National Task Force on Health Information. Ottawa, Queen’s Printer. 1991. http://www.statcan.gc.ca/pub/4220352-eng.pdf. Accessed September 2014.

[16] Canadian Institute for Health Information. About CIHI. http://www.cihi.ca/CIHI-ext-portal/internet/EN/Theme/about+cihi/cihi010702 (2014). Accessed September 2014.

[17] Institute for Clinical Evaluative Sciences. ICES: Twenty Years, 1992–2012. 2012. Available from: http://www.ices.on.ca/~/media/Files/Corporate-Reports/TWENTY_ICES_20th_Anniversary_Magazine.ashx. Accessed August 2015.

[18] Dolan D, Grainger J, MacCallum N, Creatura D, Forrester J, Shiller S (2012). The Institute for Clinical Evaluative Sciences: 20 Years and Counting. Healthcare Quarterly.

[19] Institute for Clinical Evaluative Sciences. http://www.ices.on.ca (2014). Accessed September 2014.

[20] Institute for Clinical Evaluative Sciences. Data Access Process. http://www.ices.on.ca/Data-Services/Data-Process (2015). Accessed July 2015.

[21] Marchessault G and Manitoba Centre for Health Policy. The story of the Manitoba Centre for Health Policy. Manitoba Centre for Health Policy. 2010. http://umanitoba.ca/faculties/medicine/units/community_health_sciences/departmental_units/mchp/media/Story_of_MCHP.pdf. Accessed September 2014.

[22] Manitoba Historical Society. Manitoba recipients of the Order of Canada. http://www.mhs.mb.ca/docs/people/orderofcanada.shtml (2014). Accessed September 2014.

[23] Manitoba Centre for Health Policy. About MCHP. http://umanitoba.ca/faculties/medicine/units/community_health_sciences/departmental_units/mchp/about.html (2014). Accessed September 2014.

[24] PopulationData BC. History of the health services data, and Data Linkage. http://www.popdata.bc.ca/dataaccess/history (2013). Accessed September 2014.

[25] UBC Centre for Health Services and Policy Research. History. http://www.chspr.ubc.ca/about/history. Accessed August 2015.

